# H_2_S-based fluorescent imaging for pathophysiological processes

**DOI:** 10.3389/fchem.2023.1126309

**Published:** 2023-01-27

**Authors:** Tong-Tong Jia, Yuanyuan Zhang, Ji-Ting Hou, Huawei Niu, Shan Wang

**Affiliations:** ^1^ College of Chemistry and Chemical Engineering, Luoyang Normal University, Luoyang, China; ^2^ College of Food and Bioengineering, Henan University of Science and Technology, Luoyang, China; ^3^ National Engineering Research Center of Ophthalmology and Optometry, Eye Hospital, Wenzhou Medical University, Wenzhou, China; ^4^ Key Laboratory of Intelligent Treatment and Life Support for Critical Diseases of Zhejiang Province, The First Affiliated Hospital of Wenzhou Medical University, Wenzhou, China

**Keywords:** fluorescence probe, hydrogen sulfide, pathophysiological processes, biomarker, visualization

## Abstract

Hydrogen sulfide (H_2_S), as an important endogenous signaling molecule, plays a vital role in many physiological processes. The abnormal behaviors of hydrogen sulfide in organisms may lead to various pathophysiological processes. Monitoring the changes in hydrogen sulfide is helpful for pre-warning and treating these pathophysiological processes. Fluorescence imaging techniques can be used to observe changes in the concentration of analytes in organisms in real-time. Therefore, employing fluorescent probes imaging to investigate the behaviors of hydrogen sulfide in pathophysiological processes is vital. This paper reviews the design strategy and sensing mechanisms of hydrogen sulfide-based fluorescent probes, focusing on imaging applications in various pathophysiological processes, including neurodegenerative diseases, inflammation, apoptosis, oxidative stress, organ injury, and diabetes. This review not only demonstrates the specific value of hydrogen sulfide fluorescent probes in preclinical studies but also illuminates the potential application in clinical diagnostics.

## 1 Introduction

Hydrogen sulfide (H_2_S) is the third gaseous signaling molecule found after carbon monoxide (CO) and nitric oxide (NO) (Szabo et al., 2013). Unlike other signaling molecules, H_2_S can freely penetrate the cell membrane without affecting the cell’s signaling response (Predmore et al., 2012). H_2_S is present both inside and outside the cell and is widely recognized in regulating nervous systems, cellular bioenergetics and metabolism, gene transcription and translation, vascular tone, and immune function (Cirino et al., 2022). Endogenous H_2_S is principally produced by three kinds of biological enzymes, including cystathionine *γ*-lyase (CSE), cystathionine *β*-synthase (CBS), and 3-mercaptopyruvate sulfurtransferase (3-MST) (Szabo et al., 2013; Augsburger and Szabo, 2020; Zhang et al., 2021). The physiological concentration of H_2_S ranges from 0.01 to 3 μM at the cellular level and 30–100 μM in serum (Wallace, 2007). H_2_S plays an indispensable role in physiological processes, for example, angiogenesis, neurotransmission, apoptosis, and insulin secretion (Austgen et al., 2011; Papapetropoulos, 2016; Bełtowski et al., 2018; Wang et al., 2020). Furthermore, aberrant H_2_S levels are strongly related to various pathophysiological processes, such as neurodegenerative diseases, liver cirrhosis, inflammation, and cancer (Kamoun et al., 2003; Chan and Wong, 2017; Wei et al., 2017; Bełtowski et al., 2018; Disbrow et al., 2021; Kushkevych et al., 2021; Ngowi et al., 2021). Hence, exploring validated assays for H_2_S is essential to better understand and diagnose their pathophysiological processes.

Compared with traditional imaging methods, including magnetic resonance imaging (MRI), computed tomography (CT) and ultrasound imaging (Poelma, 2016; Lim et al., 2019; Antequera et al., 2021), fluorescence imaging technology allows non-invasive detecting biomarkers with high sensitivity, quick response time and wonderful spatiotemporal resolution, which makes animal models of tracking pathology and clinical studies very attractive (Jun et al., 2020; Hanaoka et al., 2022; Qi et al., 2022; Sun et al., 2022). Fluorescence-based imaging typically uses small molecule fluorescent probes that are designed to bind/react with disease-based target biomarkers and offer measurable fluorescent signal changes for qualitative and quantitative analysis of analytes and imaging traces (Jia et al., 2022; Zhao L et al., 2022; Hou et al., 2022; Hou et al., 2020a; Gardner et al., 2021; Du et al., 2023; Li et al., 2023). Typically, these probes should exhibit wonderful sensitivity and specificity for biomarkers to guarantee their accurate detection in bio-systems (Hou et al., 2020b; He et al., 2021; Kawai et al., 2021; Ren M et al., 2021).

**FIGURE 1:**
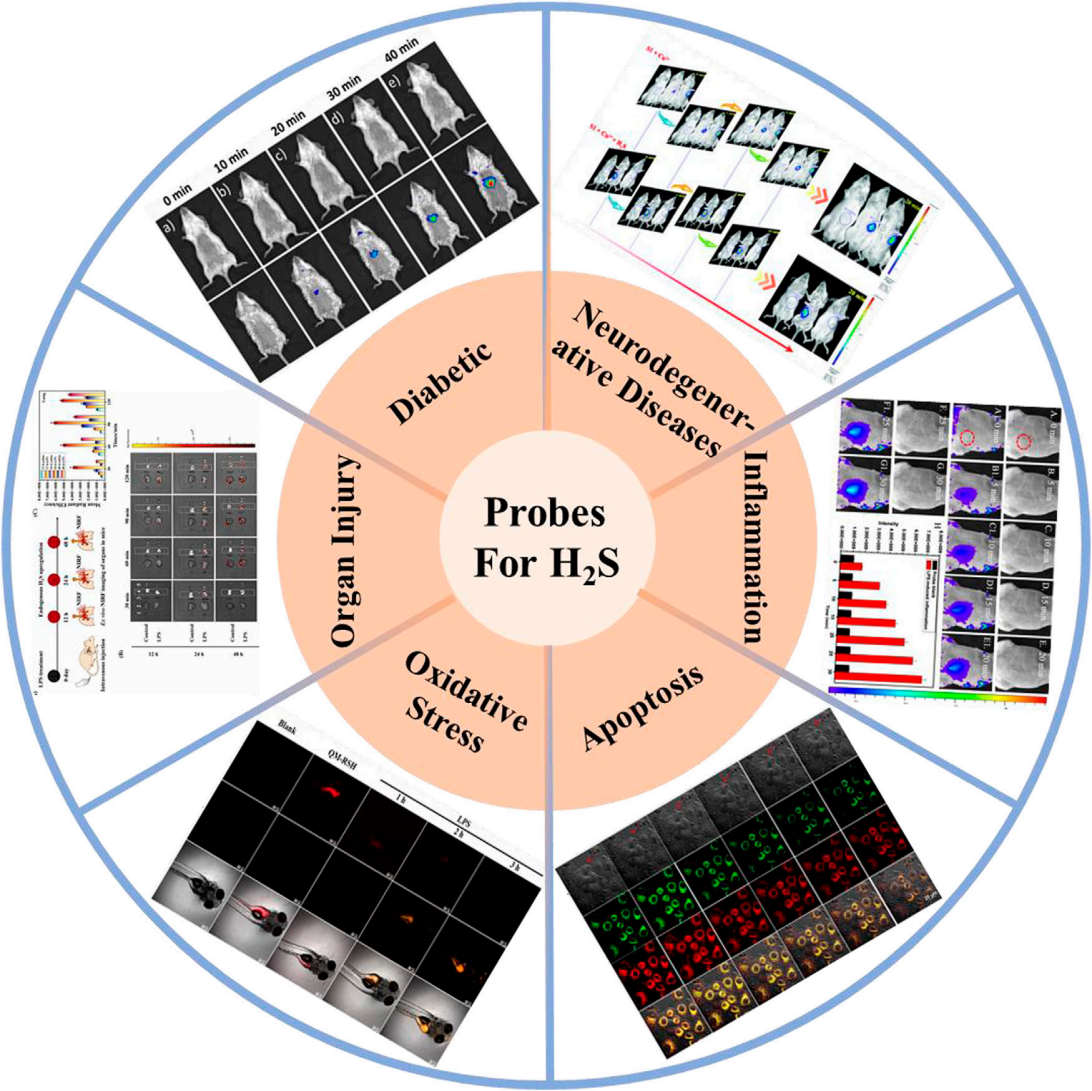
H_2_S-based small organic fluorescent probes for imaging and diagnosis of pathophysiological processes.

**FIGURE 2:**
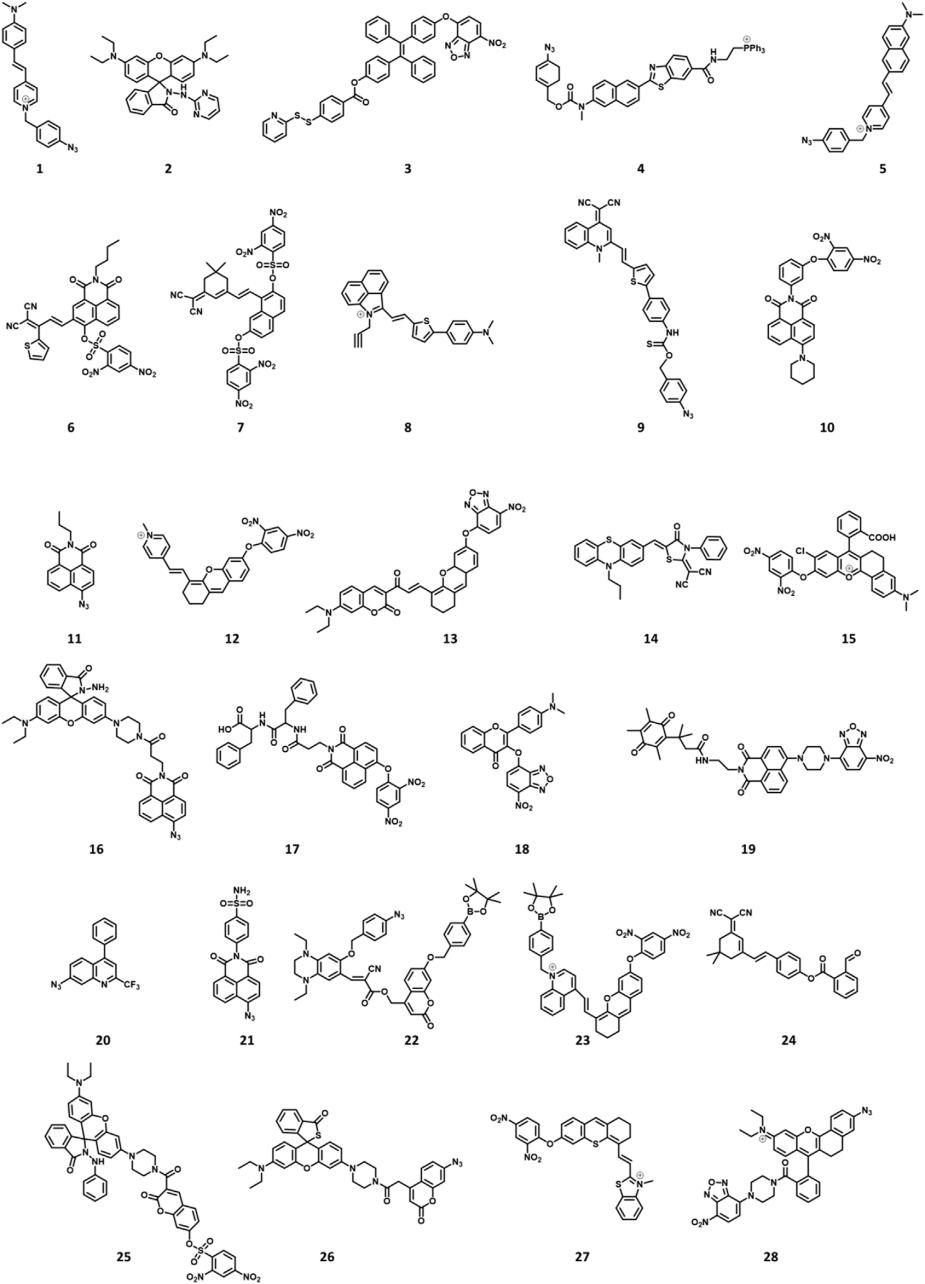
Chemical structures of H_2_S-responsive probes (1, Li et al., 2018; 2, Ma et al., 2019; 3, Ramya et al., 2022; 4, Bae et al., 2013; 5, Fang et al., 2020; 6, Shen et al., 2021; 7, Kong et al., 2021; 8, Li H et al., 2022; 9, Liang et al., 2022; 10, Ou et al., 2021; 11, Ding et al., 2022; 12, Gong et al., 2021; 13, Wang K et al., 2022; 14, Hu et al., 2021; 15, Wang WX et al., 2022; 16, Ren TB et al., 2021; 17, Singh et al., 2021; 18, Liu et al., 2022; 19, Zhang et al., 2019; 20, Zhu et al., 2020a; 21, Zhu et al., 2020b; 22, Yang et al., 2020; 23, Wang Y et al., 2022; 24, Shu et al., 2020; 25, Tang et al., 2021; 26, Jiao et al., 2018; 27, Su et al., 2022; 28, Li P et al., 2022).

This work systematically reviews the research progress of H_2_S-based fluorescent probes in pathophysiological processes imaging and classifies fluorescent probes according to pathophysiological models (neurodegenerative diseases, inflammation, oxidative stress, cell apoptosis, organ injury, and diabetes), and introduces in detail the methods, means and design ideas for constructing various disease models (Figure 1). The design tactics, optical properties, response mechanism, and potential applications of these probes are discussed (Figure 2). Furthermore, we mainly focus on the biological application and significance of H_2_S in pathophysiological pathological processes. Finally, we discuss the progress and insufficiencies of reported fluorescent probes for H_2_S-related pathophysiological processes imaging and provide our insights on how to overcome these limitations. Hence, this paper will offer new thoughts and strategies for the development of novel fluorescent probes fitting for early warning of H_2_S-related pathophysiological processes.

## 2 Design strategy for H_2_S fluorescent probes

To meet the requirements of biological applications, H_2_S-based fluorescent probes for assessing pathophysiological processes-relevant should satisfy the following requirements: 1) Noteworthy signal changes after identification of H_2_S, and prefer fluorescence enhancement change or ratiometric fluorescence changes to reduce background noise and maximize spatial resolution; 2) fluorophores with excellent photostability, high fluorescence quantum yield, and wonderful biocompatibility; 3) the ideal fluorescent probe should respond quickly to H_2_S with wonderful selectivity and sensitivity; 4) organic solvents used as little as possible, because it will damage the function of biomolecules; 5) the identification system of the probes should be silent to biomarkers, for example, HEPES (4-(2-hydroxyethyl)piperazine-1-ethanesulfonic acid) buffers react easily with hypochlorous acid (HOCl) (Xing et al., 2016).

## 3 H_2_S-based imaging of fluorescent probe pathophysiological processes models

### 3.1 Neurodegenerative diseases imaging

#### 3.1.1 Alzheimer’s disease imaging

Alzheimer’s disease (AD) is an age-related neurodegenerative disorder that can lead to dementia, usually affecting people over the age of 60 (Morales et al., 2014). The aggregation of amyloid-beta (Aβ) aggregates in the central nervous system may cause and exacerbate AD, and breaking down or stopping the formation of Aβ aggregates is a vital challenge in overcoming AD (Wood, 2017; Cao L et al., 2018; Starling, 2018; Lin et al., 2019). H_2_S donor, such as sodium sulfide (Na_2_S), reduces the generation of Aβ, thereby providing neuroprotection against Aβ aggregates and alleviating AD (Kshirsagar et al., 2020; Tabassum et al., 2020).

**FIGURE 3:**
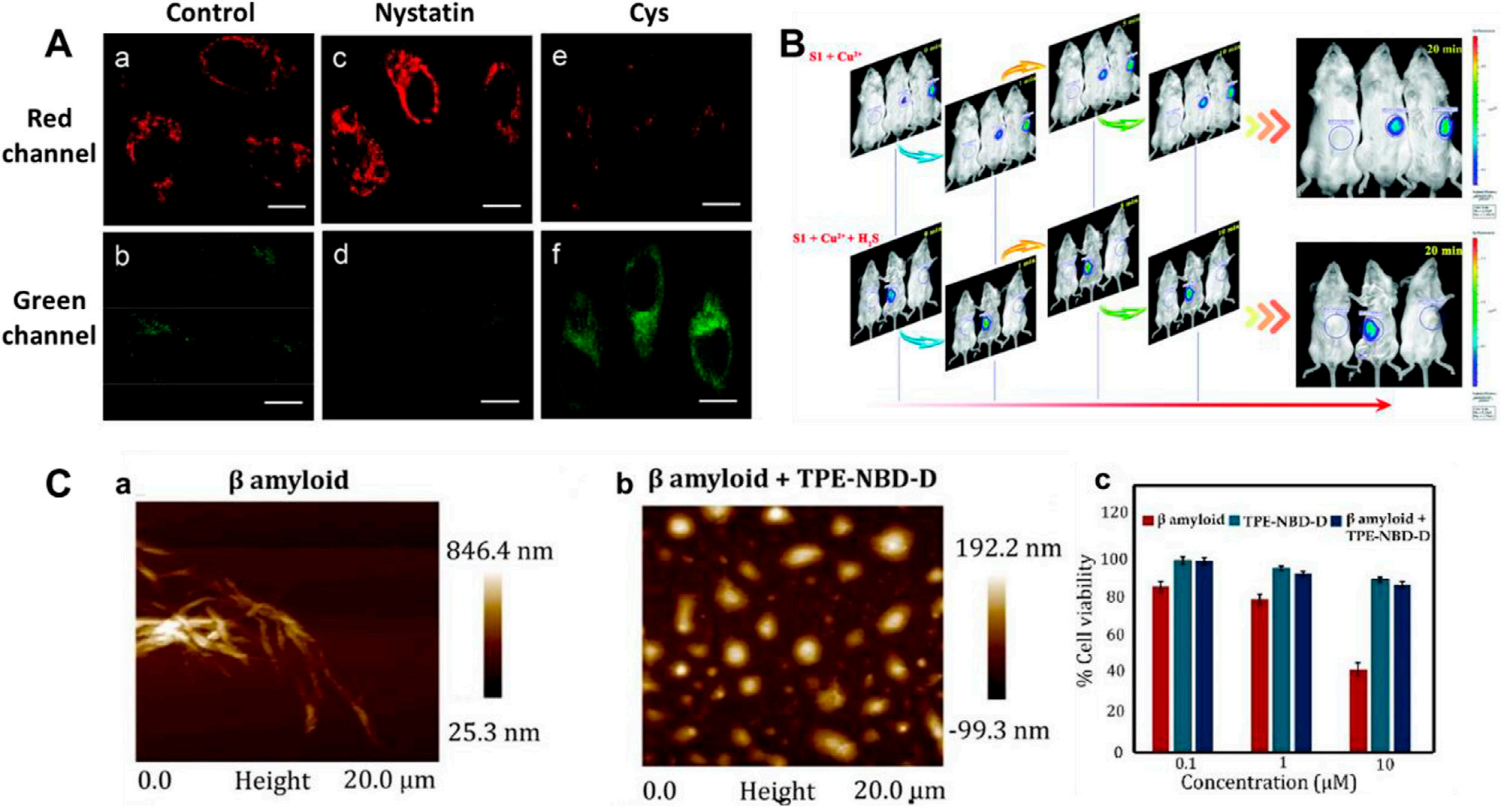
**(A)** Confocal imaging of the cross-talk influence of H_2_S and viscosity in HeLa cells using probe 1 (reproduced from (Li et al., 2018) with permission from American Chemical Society). **(B)** Time-based *in vivo* fluorescence imaging of Cu^2+^ or Cu^2+^ + H_2_S in Kunming Mice using probe 2 (reproduced from (Ma et al., 2019) with permission from the Royal Society of Chemistry). **(C)** AFM images and cytotoxicity of β sheet rich agglomerated form of Aβ_1–42_ and de-agglomerated smaller Aβ_1–42_ aggregates formed after incubation with probe 3 (reproduced from (Ramya et al., 2022) with permission from Elsevier (B. V).

Mitochondria have been used as therapeutic targets for AD (Reddy, 2009; Wang and Chen, 2016; Swerdlow, 2018). In 2018, Li et al. reported a mitochondria-targeting bifunctional fluorescent probe **1** for studying the behavior between viscosity and H_2_S in mitochondria (Li et al., 2018). A significant green fluorescence enhancement was found at approximately 510 nm after the introduction of H_2_S. Figure 3A showed the cross-talk influence of H_2_S and viscosity in cellular mitochondria: The enlargement in viscosity may result in the reduction in H_2_S, while the increase in H_2_S might lead to the decrease in viscosity. This will be helpful for understanding the pathogenesis of AD.

Cu^2+^ accumulation or H_2_S deficiency is closely related to AD (Cui W et al., 2016; Vandini et al., 2019). In 2019, Ma et al. reported an “OFF-ON-OFF” fluorescent probe **2** for reversible testing Cu^2+^ and H_2_S. Probe **2** could be used to track Cu^2+^ and H_2_S sequentially and reversibly through changes in its fluorescence signal at 580 nm. Probe **2** exhibited extremely low cytotoxicity and excellent membrane permeability. Figure 3B showed that with increasing Cu^2+^ concentration, the fluorescence in mice was significantly enhanced, while it disappeared upon the addition of H_2_S. In addition, the probe had the potential ability to disassemble Cu^2+^-induced Aβ aggregates.

Aggregation-induced emission (AIE)-based probes have wonderful features owing to their tunable emission, favorable biocompatibility, and outstanding photophysical properties (Liu and Tang, 2020; Wu and Liu, 2021; Dai et al., 2022; Li Z et al., 2022). In 2022, Ramya et al. reported a tetraphenylethylene (TPE) “double-locked” fluorescent probe **3**. The TPE fluorophore served as the core structure of AIEgen, 7-nitro-1,2,3-benzoxadiazole (NBD) acted as the recognition site for H_2_S, and the disulfide donor generated H_2_S in the presence of Cys or GSH. Probe **3** had the advantages of water solubility, low detection limit, and good selectivity for H_2_S. Figure 3C displayed that the structure of probe **3** could act as an H_2_S donor for subsequent depolymerization of Aβ_1-42_ protein, limiting the development of AD. In the presence of probe **3**, the toxic aggregated Aβ_1-42_ peptide became non-toxic disaggregated Aβ_1–42_. Fluorescent probes with a “double-lock” sequential activation strategy have higher specificity and accuracy compared to the previous “single-lock” probe strategies (Liu et al., 2019).

#### 3.1.2 Parkinson’s disease imaging

Parkinson’s disease (PD) is characterized by progressive loss of dopaminergic neurons in the substantia nigra (SN) (Hirsch et al., 1988). The first sign of cognitive impairment is memory loss, and then behavioral disturbances (Gagliardi and Vannini, 2022). It has been reported that H_2_S, as an antioxidant, has protective effects on PD by scavenging highly reactive oxygen species (ROS) as an antioxidant (Kimura and Kimura, 2004; Kimura et al., 2005). As well, overexpression of CBS or use of H_2_S donors offers neuroprotection in a 6-hydroxytryptamine (6-OHDA)-induced PD model (Yin et al., 2017; Cao X et al., 2018). Therefore, studying the pathogenesis of PD will be helpful for early therapy and intervention to slow down the progression of PD in the elderly.

**FIGURE 4:**
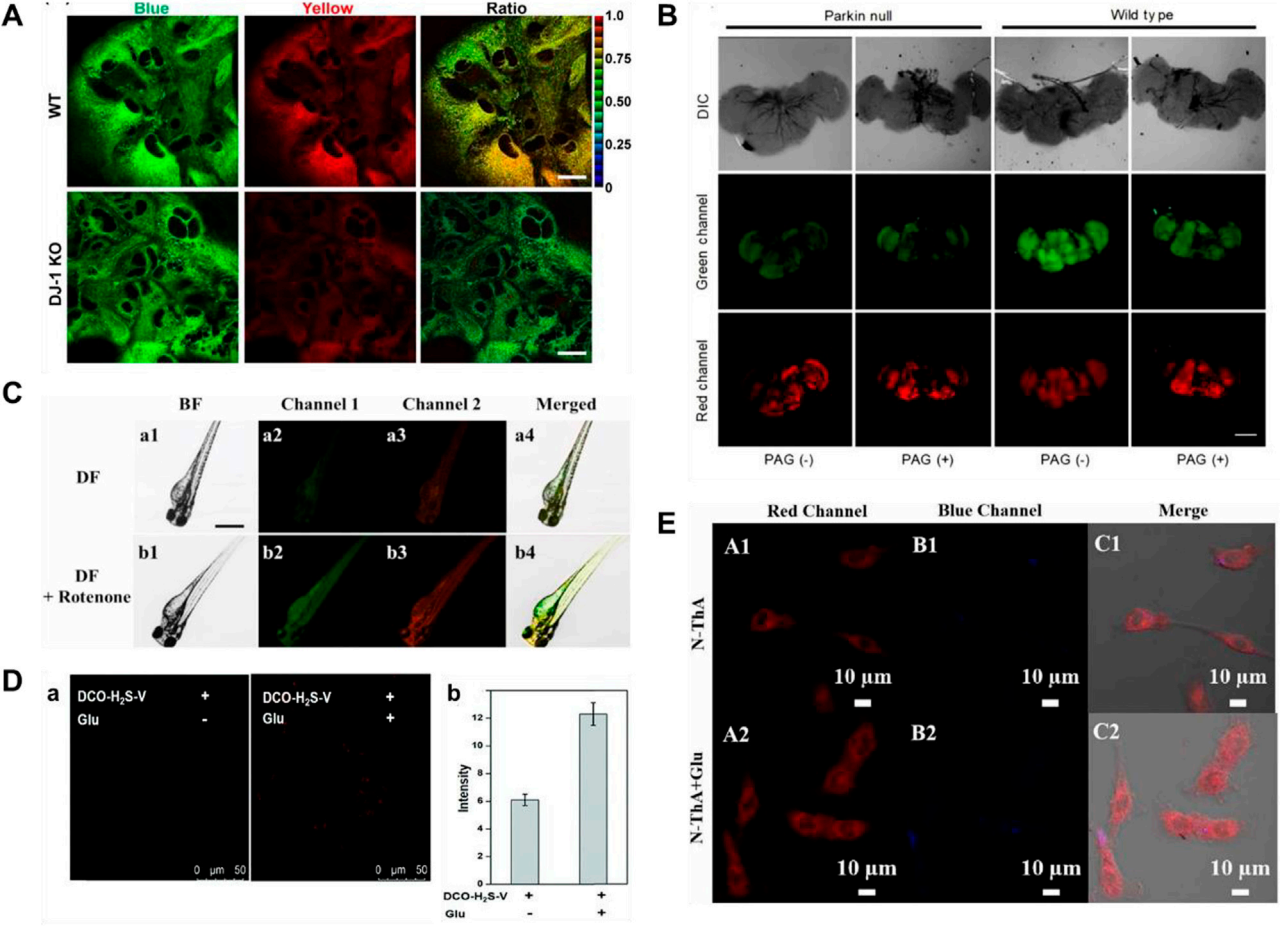
**(A)** Probe 4 displayed the correlation between CBS expression and H_2_S levels (reproduced from (Bae et al., 2013) with permission from American Chemical Society). **(B)** Fluorescence images of H_2_S and viscosity in *drosophila* brains using probe 5 (reproduced from (Fang et al., 2020) with permission from Elsevier (B. V). **(C)** Fluorescence images of viscosity and H_2_S in a zebrafish model of PD using probe **6** (reproduced from (Shen et al., 2021) with permission from Elsevier (B. V). **(D)** Fluorescence images of PC12 cells incubated with probe 7 without or with glutamate pre-treatment (reproduced from (Kong et al., 2021) with permission from the Royal Society of Chemistry). **(E)** Fluorescence images of PC 12 cells induced by Glu using probe 8 (reproduced from (Li S et al., 2022) with permission from Elsevier (B. V).

Two-photon microscopy (TPM) exhibits many wonderful merits, including larger penetration depth (>500 μm), localization of excitation, and longer observation time (Xu et al., 2020; Juvekar et al., 2021). In 2013, Kim’s group reported a ratiometric two-photon (TP) fluorescent probe (**4**) for testing H_2_S in mitochondria, in which 6-(benzo[*d*]thiazol-2′-yl)-2-(methylamino)naphthalene was used as the probe fluorophore, 4-azidobenzyl carbamate was served as the recognition site for H_2_S, and triphenylphosphonium salt could be used as the mitochondrial targeting moiety (Bae et al., 2013). When H_2_S was added, the emission peaks of probe **4** were red-shifted from 464 to 545 nm. As shown in Figure 4A, the decrease of H_2_S and decrease of CBS expression were observed in studies involving the PD gene DJ-1, in which the decrease of H_2_S in astrocytes may facilitate the progress of PD.

Mitochondria, as an important organelle, provides energy for cells, and mitochondrial dysfunction is closely related to PD (Greenamyre, 2018; Grunewald et al., 2019; Doric and Nakamura, 2021). In 2020, Fang and coworkers obtained a TP fluorescent probe **5**, using *N,N*-disubstituted unit as electron donors and pyridine cation as an electron-withdrawing group, which was used for testing mitochondrial viscosity and H_2_S (Fang et al., 2020). After different concentrations of H_2_S were introduced, the green fluorescence increased significantly. dl-Propargylglycine (PAG, a specific inhibitor of endogenous production of H_2_S)-induced PD *Drosophila* brains model had higher viscosity and lower H_2_S in mitochondria compared to the normal model (Figure 4B).

Although probe **5** has wonderful selectivity, fine sensitivity, and low detection limit for H_2_S, azide, the recognition group of the probe, can be decomposed by UV light, so false signals may be generated. In 2021, Shen and coworkers created a bifunctional near-infrared fluorescence (NIR) probe (**6**), which used dicyanoisopherone as the fluorescence core and 2,4-dinitrobenzenesulfonyl ether as the recognition group of H_2_S. Probe **6** had high photostability and a large stokes shift (110 nm). As the augment of H_2_S concentration, the fluorescence signal around 650 nm increased 20-fold. Moreover, the fluorescence signal of probe **6** around 580 nm changed with increasing viscosity. The changes in H_2_S levels and viscosity were investigated through the experiments of a zebrafish PD model induced by rotenone (a drug to reduce dopamine levels of zebrafish) (Figure 4C). The results showed that both viscosity and H_2_S increased in the zebrafish PD model.

Fluorescent probes employing a “double-lock” sequential activation strategy have higher specificity and accuracy compared to single-site release fluorescence (Liu et al., 2019). In 2021, a “double-locked” fluorescent probe **7** for monitoring H_2_S in high-viscosity systems was obtained by Kong and coworkers (Kong et al., 2021). In high-viscosity environments (the first “key”), 2,4-dinitrobenzenesulfonate group (the second “key”) in probe **7** was recognized with H_2_S, and the fluorescence signal around 630 nm was enhanced 50-fold. As shown in Figure 4D, experiments of detecting H_2_S and viscosity in glutamate (a neurotoxin)-induced PD PC 12 cell model were conducted. The results showed that the level of H_2_S as an antioxidant was upregulated to reduce oxidative stress in glutamate-induced PC12 cells.

Response time is one of the important indicators for the evaluation of probes in biological applications. As shown in Table 1, the reported H_2_S fluorescent probes for PD imaging were slow (15–120 min). In 2022, Li S et al. (2022) reported a bifunctional fluorescent probe (**8**) to detect viscosity and H_2_S in mitochondria. As viscosity gradually increased, the fluorescence signal of probe **8** around 730 nm was increased. The probe reached a response plateau after the addition of H_2_S for 8 min, with a 6-fold amplification of the fluorescence signal around 516 nm. Probe **8** was successfully applied to test the viscosity behavior of a PD model (PC-12 cells treated with glutamate), in which both H_2_S and viscosity increased in PD. As shown in Figure 4E, after injection of nystatin or glutamate in nude mouse tumor models, the red fluorescence enhanced notably with time.

**TABLE 1:** Spectroscopic properties and pathophysiological models imaging of small molecular probes for detection of H_2_S.

Probe	Pathophysiological models	LOD	λ_ex_/λ_em_ (nm)	Response time	Recognition system	Comment	Real sample	References
1	Alzheimer’s disease	0.17 μM	370/510	30 min	PBS buffer solution (pH = 7.4)	Dual-response (viscosity and H_2_S); mitochondrial targetable; increase in fl. intensity (up to 7-fold)	Living cells	Li et al. (2018)
2	Alzheimer’s disease	14.8 nM	540/580	—	PBS buffer solution (pH = 7.4, containing 50% EtOH)	Dual-response (Cu^2+^ and H_2_S)	Living cells and living mice	Ma et al. (2019)
3	Alzheimer’s disease	0.1 μM	364/480	—	HEPES buffer solution (pH = 7.4, containing 10% THF)	AIE-fluorescence; “double-locked”; increase in fl. intensity (up to 12-fold)	Living cells and living mice	Ramya et al. (2022)
4	Parkinson’s disease	0.4 μM	340/500, 420	60 min	HEPES buffer solution (30 mM, pH = 7.4, containing 100 mM KCl)	Two-photon; mitochondrial targetable probe; ratiometric I_500_/I_420_	Living cells and tissue	Bae et al. (2013)
5	Parkinson’s disease	11.66 nM	480/585	120 min	PBS buffer solution (pH = 7.4, containing 50% DMSO)	Dual-response (viscosity and H_2_S); mitochondrial targetable; increase in fl. intensity	Living cells, tissue, and *drosophila* brains	Fang et al. (2020)
6	Parkinson’s disease	79 nM	540/650	<15 min	PBS buffer solution (pH = 7.4, containing 1% DMSO)	Dual-response (viscosity and H_2_S); large stokes shift (110 nm); NIR imaging; increase in fl. intensity (up to 20-fold)	Living cells, tissue, and living zebra fishes	Shen et al. (2021)
7	Parkinson’s disease	0.1 μM	460/630	—	PBS buffer solution (pH = 7.4, containing 10% glycerol)	Dual-response (H_2_S and viscosity); “double-locked”; increase in fl. intensity (up to 63-fold)	Living cells	Kong et al. (2021)
8	Parkinson’s disease	—	385/516	8 min	PBS buffer solution (pH = 7.4, containing 30% DMSO)	Dual-response (viscosity and H_2_S)	Living cells and living mice	Li S et al. (2022)
9	Stroke	1.3 nM	450/670	40 min	PBS buffer solution (pH = 7.4, containing 80% glycerol and 2% DMSO)	NIR imaging; increase in fl. intensity (up to 25-fold)	Living cells and living mice	Liang et al. (2022)
10	Inflammation	18.8 nM	400/540	10 min	PBS buffer solution (10 mM, pH = 7.4, containing 1% DMSO)	Two-photon; increase in fl. intensity (up to 258-fold)	Living cells and tissue	Ou et al. (2021)
11	Inflammation	0.74 μM	440/561	60 min	PBS buffer solution (10 mM, pH = 7.4, containing 1% DMSO)	Two-photon; increase in fl. intensity (up to 38.1-fold)	Living cells, tissue, and living mice	Ding et al. (2022)
12	Inflammation	19 nM	530/663	3 min	PBS buffer solution (10 mM, pH = 7.4)	NIR imaging; large Stokes shift (141 nm); mitochondrial targetable; increase in fl. intensity (up to 27-fold)	Living cells, living zebra fishes, and living mice	Gong et al. (2021)
13	Inflammation	13 nM	540/699	4 min	PBS buffer solution	NIR imaging; large Stokes shift (155 nm); increase in fl. intensity (up to 75-fold)	Living cells, living zebra fishes, and living mice	Wang K et al. (2022)
14	Inflammation	1.8 μM	425/596	10 min	PBS buffer solution (20 mM, pH = 7.4, containing 30% DMF)	Colorimetric; increase in fl. intensity (up to 34-fold)	Living cells and living mice	Hu et al. (2021)
15	Inflammation	310 nM	565/620	120 s	PBS buffer solution	Mitochondrial targetable, increase in fl. intensity (up to 234-fold)	Living cells, living zebra fishes, and living mice	Wang WX et al. (2022)
16	Apoptosis	31 μM	450/540	15 min	PBS buffer solution (pH = 7.4, containing 30% DMF)	Dual-response (copper II) and H_2_S); increase in fl. intensity (up to 40-fold)	Living cells, and living zebra fishes	Ren M et al. (2021)
17	Apoptosis	—	450/550	45 min	—	Membrane permeability; specific imaging of cancer cells; increase in fl. intensity	Living cells	Singh et al. (2021)
18	Apoptosis	64 nM	−/550	30 min	PBS buffer solution (20 mM, pH = 7.4, containing 5% DMSO)	Increase in fl. intensity	Living cells	Liu et al. (2022)
19	Oxidative Stress	9 μM	−/535	120 min	PBS buffer solution (50 mM, pH = 7.4, containing 0.007% BSA, 100 μM NADH)	Dual-response (hNQO1 and H_2_S); “double-locked”; increase in fl. intensity (up to 400-fold)	Living cells	Zhang et al. (2019)
20	Oxidative Stress	0.11 μM	390/515	30 min	PBS buffer solution (10 mM, pH = 7.4, containing 20% DMSO)	Golgi targetable, increase in fl. intensity	Living cells and living zebra fishes	Zhu et al. (2020a)
21	Oxidative stress	0.10 μM	440/550	25 min	PBS buffer solution (10 mM, pH = 7.4)	Golgi targetable, increase in fl. intensity	Living cells and living zebra fishes	Zhu et al. (2020b)
22	Oxidative stress	0.058 μM	325/627, 413	80 min	HEPES buffer (20.0 mM, pH = 7.4, containing 1.0 mM CTAB)	Dual-response (H_2_O_2_ and H_2_S); two increased fluorescence signals	Living cells and living zebra fishes	Yang et al. (2020)
23	Oxidative stress	44.6 nM	460/550	10 min	PBS buffer solution (25 mM, pH = 7.4, containing 30% CH_3_CN)	Dual-response (H_2_O_2_ and H_2_S); mitochondrial targetable; “double-locked”; increase in fl. intensity	Living cells and living zebra fishes	Wang Y et al. (2022)
24	Oxidative stress	39.1 nM	480/560, 650	12 min	PBS buffer solution (10 mM, pH = 7.4, containing 10% DMSO)	NIR imaging; large Stokes shift (150 nm); endoplasmic reticulum targetable; ratiometric I_650_/I_560_	Living cells and living zebra fishes	Shu et al. (2020)
25	Oxidative stress	17.16 nM	400/464	—	PBS buffer solution (10 mM, pH = 7.4, containing 20% CH_3_CN)	Dual-response (ONOO^−^ and H_2_S); increase in fl. intensity	Living cells	Tang et al. (2021)
26	Organ injury	192.1 nM	360/445	15 min	PBS buffer solution (50 mM, pH = 7.4, containing 10% DMF)	Dual-response (HClO and H_2_S); two-photon; increase in fl. intensity	Living cells and tissue	Jiao et al. (2018)
27	Organ injury	0.09 μM	720/787	120 min	PBS buffer solution (20 mM, pH = 7.4, containing 5% DMSO)	NIR imaging; increase in fl. intensity (up to 52-fold)	Living cells, living mice , and lung organs	Su et al. (2022)
28	Diabetes	33 nM	600/633	—	PBS buffer solution (20 mM, pH = 7.4)	NIR imaging; “double-locked”; increase in fl. intensity	Living cells and living mice	Li Z et al. (2022)

#### 3.1.3 Stroke imaging

**FIGURE 5:**
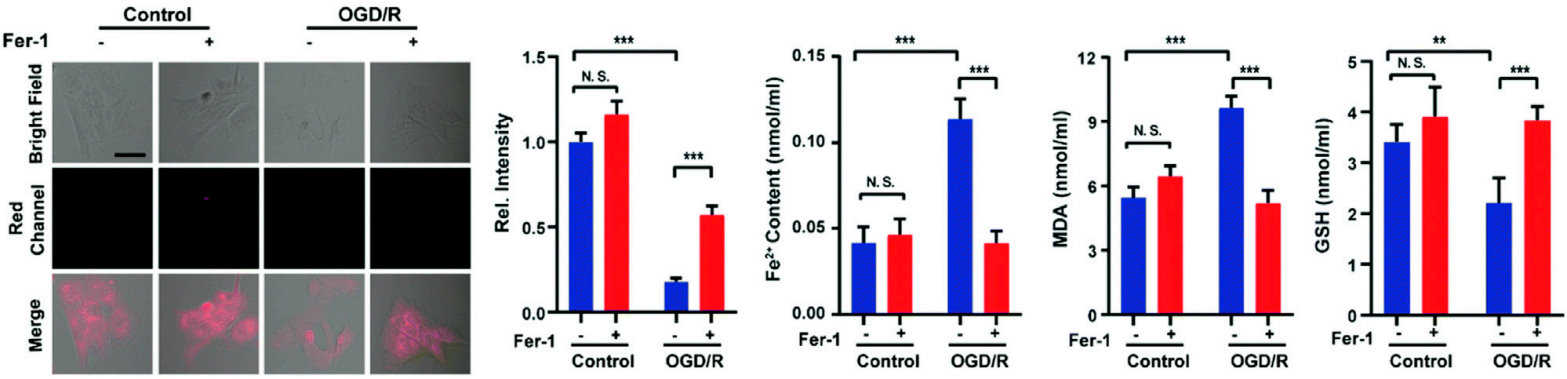
Probe 9 for H_2_S: High-fidelity ferroptosis evaluation in cells during the stroke (reproduced from (Liang et al., 2022) with permission from the Royal Society of Chemistry).

Ferroptosis (iron-dependent oxidative stress) is closely associated with cancer, neurodegenerative diseases, ischemia-reperfusion injury, etc., and detecting its pathological processes is vital for disease prognosis and treatment (Qiu et al., 2020; Yu et al., 2021; Zhao et al., 2021; Lei et al., 2022; Zhao Y et al., 2022). In 2022, Liang and colleagues reported a NIR fluorescent probe (**9**) with H_2_S triggering and H_2_S releasing properties. Azidobenzene served as the H_2_S recognition site and was linked to the fluorophore *via* thiocarbamate (H_2_S precursor). When probe **9** reacted with H_2_S, carbonyl sulfide (COS) was released by 1,6-elimination reactions, and then H_2_S was released catalyzed by carbonic anhydrase (CA). In glycerol, probe **9** had a strong fluorescence signal at 646 nm. As the H_2_S concentration increased, the fluorescence signal around 670 nm increased approximately 25-fold. Moreover, the relationship between oxygen-glucose deprivation/re-oxygenation (OGD/R) and ferroptosis was studied with PC12 cells. Figure 5 showed that the process of cell ischemia-reperfusion was accompanied by ferroptosis and H_2_S depletion.

### 3.2 Inflammation imaging

Inflammation mainly includes two categories, infectious and non-infectious, manifested as swelling, redness, pain, fever, dysfunction, etc (Fontaine et al., 2016). Inflammation is usually beneficial to biological systems, and it is an automatic defense response of biological systems. However, sometimes inflammation can be harmful to tissues and organisms. For example, out-of-control inflammation can be responsible for cardiovascular and cerebrovascular diseases, fibrosis, and cancer (Capuron et al., 2008; Mantovani et al., 2008; Lim, 2018; Mack, 2018; Weiss, Ganz, and Goodnough, 2019). These diseases and inflammation are always mutually reinforcing (Jiang et al., 2019; Majd, Saunders, and Engeland, 2020; Liberale et al., 2022). Therefore, accurate diagnosis at the initial stages of inflammation and preventing the further development of inflammation into more severe diseases is important. H_2_S can achieve anti-inflammatory effects by inhibiting the production of inflammatory cytokines, and its overexpression *in vivo* has been considered as a biomarker of all kinds of inflammation. So, it is vital to investigate the behaviors or relationships between H_2_S and inflammation in biological systems.

**FIGURE 6:**
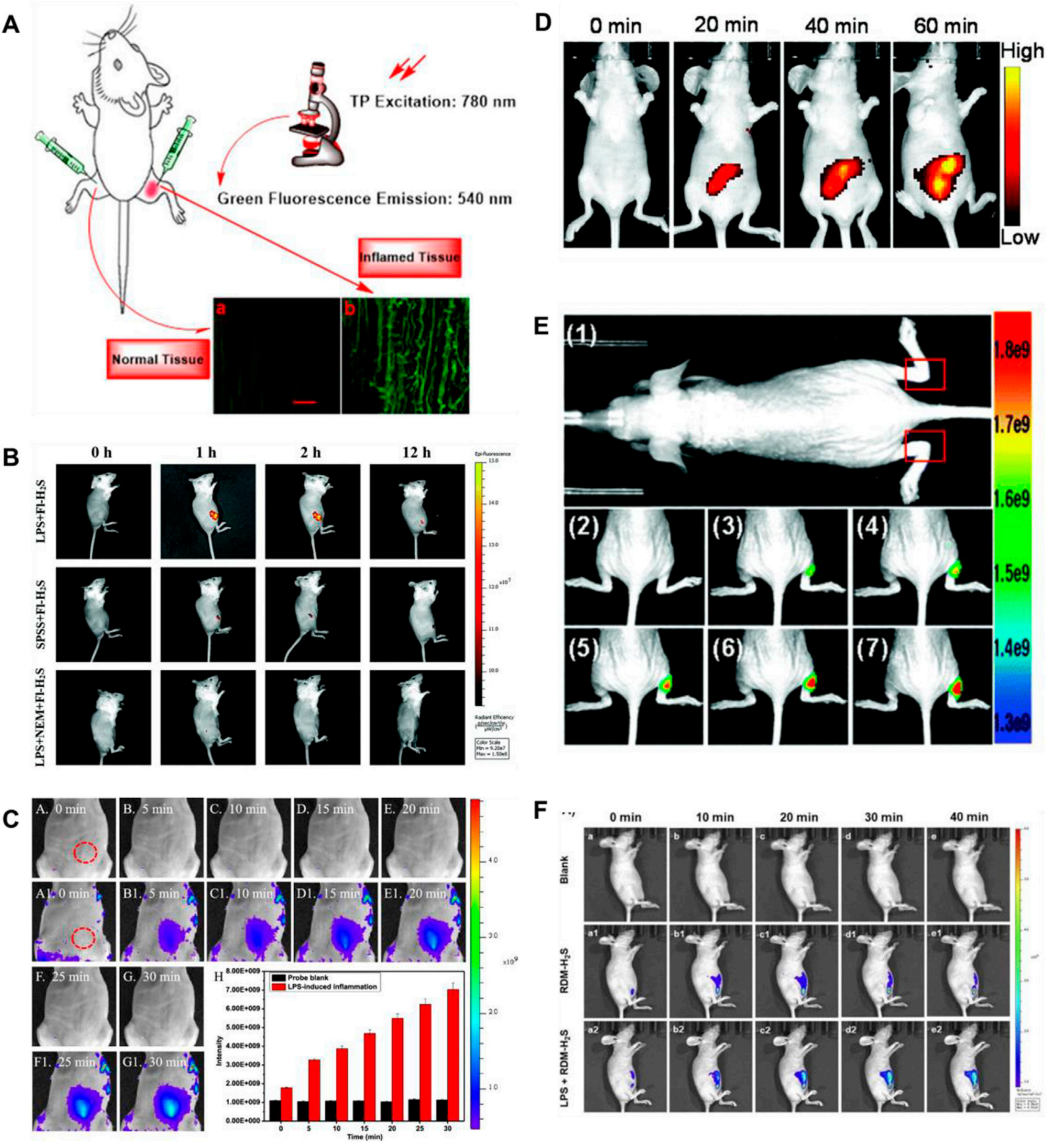
**(A)** Images of a frozen inflamed and normal tissue slice from Kunming mouse using probe 10 (reproduced from (Ou et al., 2021) with permission from Elsevier (B. V). **(B)** Time-dependent fluorescence images of air pouch inflammation in a female nude mouse before and after subcutaneous injection of probe 11 (reproduced from (Ding et al., 2022) with permission from the Royal Society of Chemistry). **(C)** Imaging of H_2_S during the LPS-induced inflammation in mice using probe 12 (reproduced from (Gong et al., 2021) with permission from American Chemical Society). **(D)** Fluorescence images of H_2_S in the inflammation mice model using probe 13 (reproduced from (Wang Y et al., 2022) with permission from the Royal Society of Chemistry). **(E)** Fluorescence images of H_2_S generation in an inflammation model in live nude mice using probe 14 (reproduced from (Hu et al., 2021) with permission from the Royal Society of Chemistry). **(F)** Fluorescence imaging of probe 15 in LPS-induced inflammatory processes in living mice (reproduced from (Wang WX et al., 2022) with permission from Elsevier (B. V).

Lipopolysaccharide (LPS), as a dominating cell surface component of Gram-negative bacteria, can be used for bioimaging to induce cellular inflammation models (Lykhmus et al., 2016). In 2021 and 2022, Ou et al. (2021), Ding et al. (2022) fabricated TP fluorescence probes (**10**, **11**) for H_2_S imaging in inflammatory models, respectively. Probe **10** consisted of naphthalimide derivative as a fluorophore and 4- dinitrophenyl ether (DNB) as a recognition group. When H_2_S existed, probe **10** exhibited amazing fluorescence enhancement (258-fold) at 540 nm. Figure 6A showed that compared with normal tissues, the inflamed tissues had a significant fluorescence signal augmentation in the green channel. Probe **11** consisted of azide and a fluorophore of naphthylimide. When H_2_S was introduced, the fluorescence signal around 561 nm was enhanced 38.1-fold. In addition, probe **11** exhibited excellent TP fluorescence properties in cells and liver tissues, penetrating to depths of 126 μm in liver tissue. As shown in Figure 6B, the experiment of the LPS-induced air pouch inflammation model was conducted to observe the development of inflammation and the behavior of H_2_S.

In 2021 and 2022, Gong’s group and Wang’s group fabricated NIR mitochondrial-targeting fluorescent probes (**12**, **13**) for H_2_S imaging in inflammatory models, respectively. In probe **12**, the pyridium unit (positively charged) acted as a mitochondria-targeting group and dinitrophenyl (DNP) ether as an H_2_S recognition group. When H_2_S was added, a fluorescence-enhancing signal around 663 nm appeared. Probe **12** had the advantages of wonderful water solubility, fast response (<3 min), and large Stokes shift (141 nm). As shown in Figure 6C, changes in H_2_S concentration were performed during LPS-induced inflammation in mice. The results suggested that more H_2_S could be produced during inflammation. Probe **13** consisted of a NIR fluorophore and a recognition group (NBD). After H_2_S was introduced, probe **13** showed a remarkable enhancement (75-fold) in fluorescence signal at 699 nm. Probe **13** exhibited a large Stokes shift (155 nm), quick response (4 min), and wonderful selectivity for H_2_S. Probe **13** could detect exogenous and endogenous H_2_S in live cells and zebrafish, respectively. Figure 6D showed that probe **13** was used to monitor H_2_S fluctuations in LPS-induced inflammatory cells and mice.

Colorimetric detection can be recognized by the naked eye. In 2021, Hu et al. (2021) developed a phenothiazine-based colorimetric fluorescence probe (**14**) to selectively detect H_2_S in an LPS-induced inflammation mouse model. Probe **14** was based on a donor–π–acceptor (D–π–A) structure that coupled phenothiazine to rhodanine derivative *via* a carbon-carbon double bond. During the probe’s identification of H_2_S, the fluorescence signal around 596 nm showed a significant increase (34-fold). Probe **14** was able to visualize exogenous and endogenous H_2_S *in vitro* and *in vivo* (zebrafish and nude mice). Figure 6E showed that visualization of the production of H_2_S in inflammatory models has been realized by probe **14**.

Rhodamine dyes are attracting attention for their wonderful photostability, long emission wavelength, convenient synthesis, and high quantum yield (Rajasekar, 2021). In 2022, Wang and coworkers created a mitochondrial-targeting fluorescent probe **15** to test the changes in H_2_S concentration. The fluorescence intensity around 620 nm progressively augmented about 234-fold with increasing H_2_S concentration. Probe **15** had some wonderful features of fast response (120 s), low detection limit (310 nM), and excellent sensitivity. Probe **15** could monitor exogenous and endogenous H_2_S in HeLa cells and zebrafish, respectively. Probe **15** could be used to visually detect H_2_S in LPS-induced mouse inflammation experiments (Figure 6F). And probe **15** was appropriate for testing the behavior of H_2_S in human plasma samples.

### 3.3 Apoptosis imaging

Apoptosis is caused by pathological and physiological conditions triggered by extracellular death receptor ligation or DNA damage and/or cytoskeletal disruption (Akçapınar et al., 2021). The intrinsic way of apoptosis is triggered by the cell’s response to injury, while the external way is triggered by cell-stimulated death receptors of the immune system (Sica et al., 1990; Oppenheim et al., 2001). When caspase 3 is activated, both pathways converge, leading to cell death (D’arcy, 2019). Timely monitoring of apoptosis is helpful for early warning and therapy of related pathophysiological processes and the continuous assessment of drug effectiveness. H_2_S has been found to protect cells: H_2_S can prevent Abeta-induced neuronal apoptosis by diminishing mitochondrial translocation of phosphatase and tensin homolog deleted on chromosome ten (PTEN) (Cui Z et al., 2016); H_2_S can restrain cell apoptosis and protect bronchial epithelium in a mouse model of allergic inflammation (Mendes et al., 2019); H_2_S improves LPS-induced memory disorder in mice by decreasing apoptosis, oxidation, and inflammatory effects (Kshirsagar et al., 2021). However, H_2_S can also promote apoptosis: H_2_S contributes to LPS-induced osteoblast apoptosis by restraining the AKT/NF-κB signaling pathway (Wang et al., 2020); H_2_S, which releases whey protein derivatives, induces apoptosis through extrinsic and intrinsic pathways (Li et al., 2020). Therefore, the exact relationship between H_2_S and apoptosis needs to be further studied.

**FIGURE 7:**
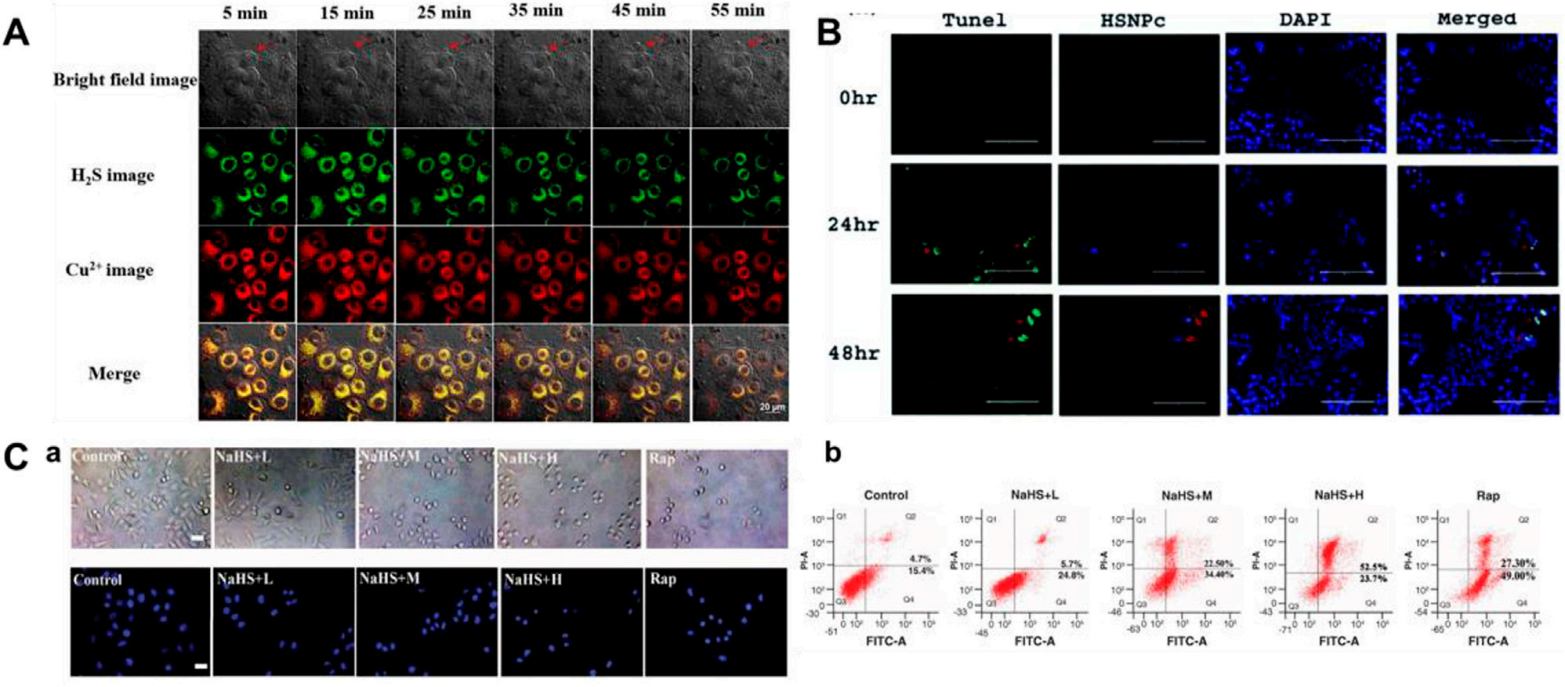
**(A)** Simultaneous fluorescent images of copper (II) ions and H_2_S in HeLa cells stained with probe 16 and treated with CuSO_4_ and NaHS at different times (reproduced from (Ren M et al., 2021) with permission from Elsevier (B. V). **(B)** Determination of apoptosis by TUNEL assay using probe 17 (reproduced from (Singh et al., 2021) with permission from the Royal Society of Chemistry). **(C)** Apoptosis induced by H_2_S leads to decrease in cell viability using probe 18 (reproduced from (Liu et al., 2022) with permission from Newlands Press).

Cu/NaHS significantly reduced the Menkes copper transport (ATP7A) protein levels, promoted intracellular Cu accumulation, and resulted in increased Cu cytotoxicity (Goto et al., 2020). Therefore, continuous detection of H_2_S and Cu^2+^ is helpful to understand their interaction. In 2021, a bifunctional fluorescent probe (**16**) for testing H_2_S and Cu^2+^ in different channels in live cells and zebrafish was reported by Ren and colleagues. Naphthalimide and rhodamine were used as probe fluorophores, and azide and hydralazine were selected as recognition sites for H_2_S and Cu^2+^. The fluorescence intensity augmented 40-fold and 31-fold in response to H_2_S and Cu^2+^, respectively. Probe **16** allowed simultaneous fluorescence imaging of H_2_S and Cu^2+^ in cells, enabling visualization of H_2_S-enhanced Cu^2+^ cytotoxicity (Figure 7A).

In 2021, Singh et al. fabricated a naphthalimide-based bifunctional fluorescent probe **17** for detecting H_2_S, which was made up of a peptide-naphthalimide fluorophore and an H_2_S sensing moiety. When H_2_S was introduced, the morphology of probe **17** showed the combination of fibrous “bushes” with bright yellow fluorescence. Probe **17** had the ability of cancer cell imaging and induction of apoptosis in the meantime, which could be a good candidate for the theranostic agent (Figure 7B).

Because of its fascinating optical properties, including large Stokes shift, “turn-on” fluorescence, relatively high quantum yield, and good photostability, 3-hydroxyflavone has been widely concerned by researchers (Sedgwick et al., 2018; Wang, Lai, Qiu and Liu, 2019; Doric and Nakamura, 2021). In 2022, Liu et al. (2022) created a fluorescent probe **18** based on excited state intramolecular proton transfer (ESIPT) for testing H_2_S. The probe consisted of 3-hydroxyflavone and 4-Chloro-7-nitro-1,2,3-benzoxadiazole (NBD-Cl, H_2_S-specific recognition unit). When H_2_S existed, 3-hydroxyflavone formed a ketone tautomer and released fluorescence at 550 nm. Figure 7C showed the behavior of different concentrations of H_2_S on the apoptosis of MCF-7 cells.

### 3.4 Oxidative stress imaging

The imbalance between oxidants and antioxidants is beneficial to oxidants and can cause damage, known as oxidative stress (Sies, 1997). Oxidants are normal products of aerobic metabolism, but they can be produced at a higher rate under pathophysiological conditions. If left unchecked, oxidative stress can lead to damage to DNA, proteins, and lipids, and ultimately cell death (Greenwood and Witney, 2021). H_2_S has been proven to influence cellular redox through multiple mechanisms, such as ROS scavenging, protein modification, mitochondria, and respiratory oxidation (Pal, Bandyopadhyay, and Singh, 2018; Scammahorn et al., 2021). Furthermore, some suborganelles are related to oxidative stress, for example, the Golgi apparatus actively participates in the stress response, and when larger than the stress threshold, the Golgi apparatus can simultaneously activate cell repair and apoptosis mechanisms (Hicks and Machamer, 2005; Wlodkowic, Skommer, Mcguinness, Hillier, and Darzynkiewicz, 2009); H_2_S can effectively decrease endothelial-mesenchymal conversion by restraining ER stress (Ying et al., 2016). Therefore, tracking H_2_S behaviors in different organelles is crucial for the research and treatment of related diseases or pathophysiological processes.

**FIGURE 8:**
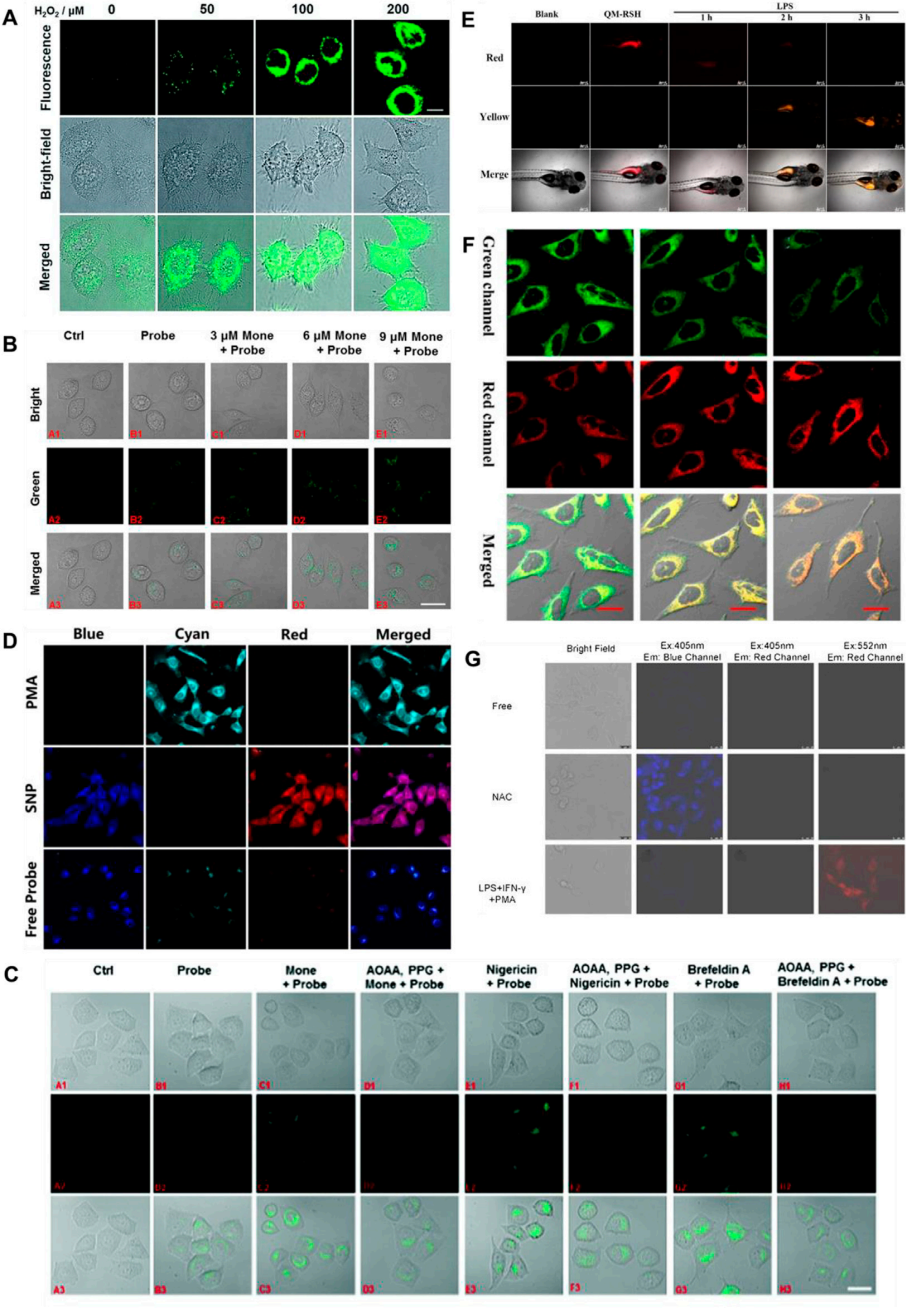
**(A)** Confocal microscopy images for concentration-dependent H_2_O_2_-induced fluorescence in living HeLa cells using probe 19 (reproduced from (Zhang et al., 2019) with permission from the Royal Society of Chemistry). **(B)** Golgi stress response experiments in cells using probe 20 (reproduced from (Zhu et al., 2020a) with permission from American Chemical Society). **(C)** Fluorescence imaging of probe 21 after stimulating cells with only probe 22, Mone, aminooxyacetic acid (AOAA)/photoplethysmographic (PPG) Mone, nigericin, AOAA/PPG/igericin, brefeldin A, and AOAA/PPG/brefeldin A, respectively (reproduced from (Zhu et al., 2020b) with permission from the Royal Society of Chemistry). **(D)** Confocal fluorescence images of endogenous H_2_O_2_/H_2_S in living HeLa cells using probe 22 (reproduced from (Yang et al., 2020) with permission from American Chemical Society). **(E)** Fluorescence imaging H2S in inflammation response zebrafish using probe 23 (reproduced from (Wang K et al., 2022) with permission from Elsevier (B. V). **(F)** Confocal imaging of H_2_S during ER stress with probe 24 (reproduced from (Shu et al., 2020) with permission from American Chemical Society). **(G)** HUEVC cells imaging endogenous ONOO^−^ and H_2_S using probe 25 (reproduced from (Tang et al., 2021) with permission from Elsevier (B. V).

H_2_S and human NAD(P)H:quinine oxidoreductase 1 (hNQO1), as latent cancer biomarkers, were able to participate in cell redox homeostasis (Park, et al., 2021). In 2019, Zhang et al. (2019) developed a dual biomarker (H_2_S and hNQO1)-triggered fluorescent probe to reveal the synergistic antioxidant effect under oxidative stress. Quinone propionic acid (Q_3_PA) and NBD served as hNQO1 and H_2_S recognition units, and coumarin and naphthalimide acted as fluorophores of probe **19**, respectively. The strategy of dual reaction and dual quenching was formed, which improved the sensitivity and selectivity of the probe. When H_2_S existed, the fluorescence signal of probe **19** was remarkably enhanced (400-fold) at 535 nm. In addition, the probe could simultaneously test the endogenous H_2_S and hNQO1 activities in organic systems. Figure 8A showed that HeLa cells could induce the production of endogenous H_2_S under the existence of exogenous hydrogen peroxide (H_2_O_2_), that is, H_2_S played a synergistic antioxidant role under oxidative stress.

The Golgi stress response is activated when Golgi function is inadequate compared to cellular demands (Gao, et al., 2021). Golgi apparatus provides cytoprotection by moderating the synthesis and metabolism of bioactive molecules in response to conventional stress (Paul, et al., 2014; Hirayama, et al., 2019). In 2020, Zhu et al. (2020), Shu et al. (2020) reported Golgi-targeted fluorescent probes (**20**, **21**) detecting H_2_S, respectively. In probe **20**, 4-CF_3_-substituted 7-aminoquinoline was used as fluorophore, and azide was elected as the specific identification group of H_2_S. The introduction of trifluoromethyl into the quinoline structure facilitated the entry of the probe into the Golgi apparatus through the membrane barrier. With the H_2_S concentration increased, the fluorescence signal around 515 nm was augmented. As shown in Figure 8B, probe **20** has achieved *in situ* display of H_2_S generation under monensin-induced Golgi pressure. In probe **21**, 1,8-naphthalimide was used as the fluorophore, azide was used as the identification group of H_2_S, and phenylsulfonamide was used as the targeting group of the Golgi apparatus. When H_2_S was introduced, the fluorescence signal was remarkably enhanced at 550 nm. Furthermore, Figure 8C showed probe **21** could be seen as a chemical method to detect the behavior of H_2_S *in situ* during Golgi stress, thus confirming that H_2_S could be used as a biomarker to investigate Golgi stress.

Intracellular H_2_S and H_2_O_2_ are closely associated with maintaining cellular homeostasis, and their levels directly reflect the degree of oxidative stress and disease (Kimura and Kimura, 2004; Kimura, et al., 2009). In 2020, Yang et al. (2020) fabricated a fluorescent probe **22** for testing dynamic H_2_O_2_/H_2_S redox processes in organisms. Phenylboronate and azide moieties served as recognition units for H_2_O_2_ and H_2_S, respectively. Under the existence of H_2_O_2_, the fluorescence intensity around 413 nm declined, while the fluorescence around 486 nm enhanced remarkably. When H_2_S was added, two fluorophores (HCB and TQC) were released, and the fluorescence at 413 and 627 nm were emitted, respectively. Figure 8D showed phorbol 12-myristate 13-acetate (PMA)-induced stress experiments, in which cells produced H_2_O_2_ and reduced H_2_S. In 2022, Wang and colleagues obtained a NIR fluorescence probe activated by H_2_O_2_ to monitor the changes in H_2_S during oxidative stress. When H_2_O_2_ was present, the fluorescence signal of probe **23** blue-shifted from 700 to 550 nm after recognizing H_2_S. Probe **23** could monitor the changes in H_2_S during the oxidation-triggered oxidative stress process in cells and zebrafish. Figure 8E showed that the probe evaluated the up-regulation of H_2_S levels based on oxidative stress by H_2_O_2_/PMA.

The endoplasmic reticulum (ER) plays a critical role in protein synthesis, folding, distribution, and storage of calcium ions (BÁnhegyi, et al., 2007; Pagliassotti, et al., 2016). ER stress can result in autophagy and even cell death, which is bound up with serious diseases or pathophysiological processes (Holczer, et al., 2018). In 2020, Shu et al. (2020) reported an ER-targeted ratiometric fluorescent probe for detecting H_2_S in organism systems. Probe **24** was composed of dicyanoisophorone analogue with a large Stokes shift and *o*-carboxybenzaldehyde as the specific recognition group of H_2_S. H_2_S reacted with the aldehyde group in the probe through nucleophilic addition, emitting fluorescence at 650 nm. The probe had good selectivity, large Stokes shift (150 nm). Figure 8F showed that the probe observed the endogenous changes in H_2_S under tunicamycin-induced endoplasmic reticulum stress.

Abnormal metabolism of organisms produces high concentrations of active carbonyl substances, leading to carbonyl stress, which leads to cell injury or cell apoptosis (Bordoni, et al., 2006). Therefore, the development of tools to image carbonyl stress is essential to decrease its damage and explore new drug treatments or reduce carbonyl stress. In 2021, a visualized fluorescent probe (**25**) for monitoring the protective effect of endogenous H_2_S during carbonyl stress in endothelial cells was developed by Tang and colleagues. The probe had dual fluorophores (rhodamine and coumarin fluorophores) and dual recognition sites (phenylhydrazine and 2,4-dinitrobenzenesulfonyl ether) to achieve the purpose of dual recognition of H_2_S and ONOO^−^, and the fluorescence signals of rhodamine and coumarin would not interfere with each other (>100 nm). When H_2_S and ONOO^−^ were introduced, the probe showed remarkable increases in fluorescence signal around 464 and 570 nm, respectively. Probe **25** enabled endogenous H_2_S and ONOO^−^ imaging in different channels. Figure 8G showed that probe **25** was suitable for visualizing the protective effect of endogenous H_2_S during carbonyl stress.

### 3.5 Organ injury imaging

H_2_S is synthesized in almost all organ systems (Kasinath et al., 2018). H_2_S has been proven to protect against organ damage, including liver damage, heart damage, kidney damage, etc (Tan et al., 2011; Wang et al., 2012; Kasinath, 2014). For example, in acute or chronic kidney disorders, H_2_S generation from the renal cells is decreased (Koning et al., 2015; Lobb et al., 2015; Cao and Bian, 2016; Cao et al., 2019); Endogenous and exogenous H_2_S reduces myocardial damage and improves cardiac function (Johansen et al., 2006; Wu et al., 2021); Decreased levels of endogenous H_2_S in the brain were associated with increased lesion volume and mortality after traumatic brain injury (TBI) (Zhang et al., 2013); H_2_S prevents LPS-induced acute lung injury (ALI) by restraining synergistic pro-inflammatory and oxidative reactions of stress proteins, mitogen-activated protein kinases (MAP kinases), and ROS signaling pathways (Zimmermann et al., 2018). Therefore, the development of sensitive probes for *in vivo* imaging of H_2_S is critical for exploring H_2_S biology and the diagnosis of organ injury.

**FIGURE 9:**
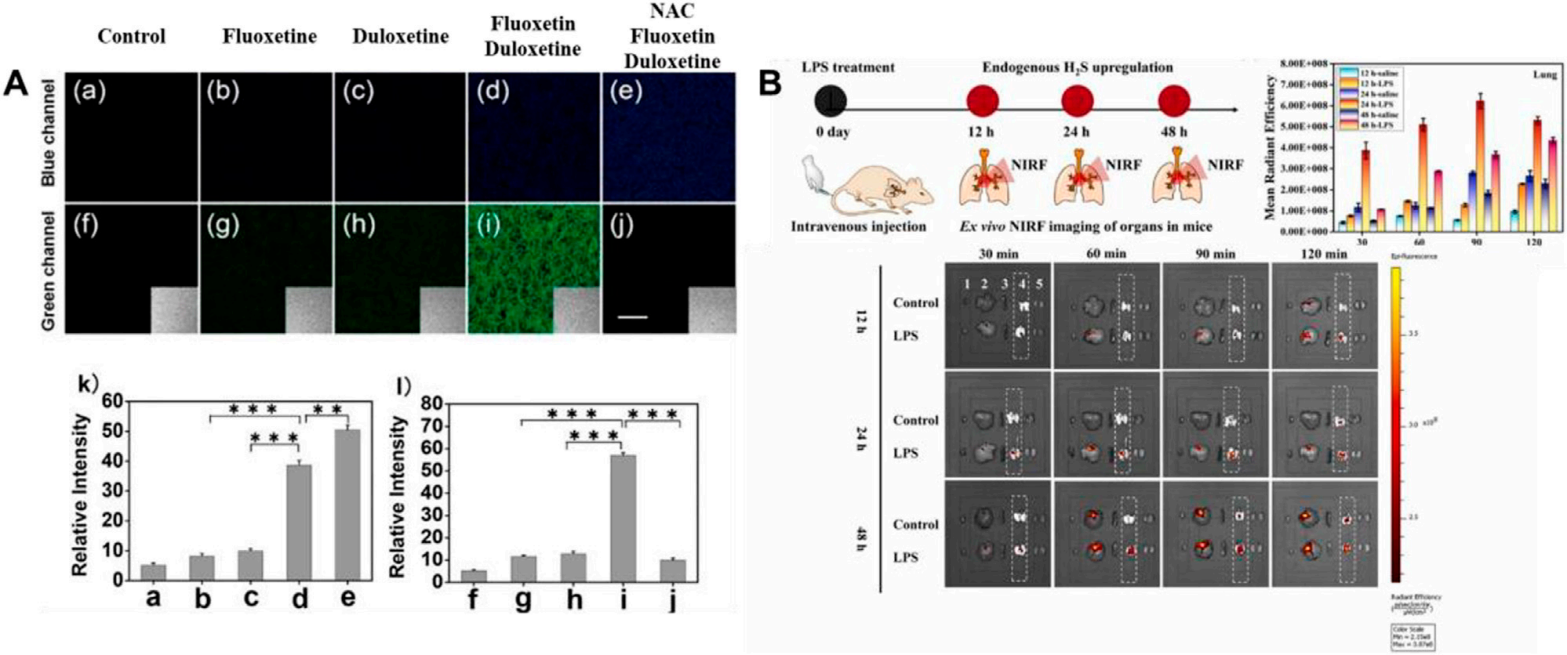
**(A)** TPM imaging of endogenous H_2_S and HClO in RAW264.7 cells upon drug treatment using probe 26 (reproduced from (Jiao et al., 2018) with permission from American Chemical Society). **(B)** Schematic illustration of probe 27 reporting the H_2_S upregulation process in ALI mice’s lungs (reproduced from (Su et al., 2022) with permission from Elsevier (B. V).

In 2018, Jiao’s group developed a TP fluorescent probe **26**, which was used to explore the potency of HClO as an indicator of drug-induced liver injury (DILI) and the detoxification of N-acetylcysteine (NAC) mediated by H_2_S. The probe was linked by 7-amino coumarin and rhodamine B *via* piperazine. When HClO or H_2_S existed, the fluorescence signal was remarkably enhanced at 580 or 445 nm. In this process, the recovery of the D-π-A structure induced by azide reduction of H_2_S and the ring opening induced by HClO were carried out separately, so that H_2_S and HClO did not generate signals that interfered with each other. As shown in Figure 9A, DILI induced by antidepressants such as duloxetine and fluoxetine and their remission were assessed at the cellular and tissue levels, respectively. The data showed that only after combined administration of the drugs, a significant increase of HClO and significant liver injury were found. At the same time, NAC pretreatment led to an increase in endogenous H_2_S levels, which was helpful in the remission of DILI.

Hemicyanine dyes have great potential in the research of small animal imaging and disease modeling owing to their emission in the NIR regions, convenient synthesis, and wavelength tunability (Li H et al., 2022). In 2022, a NIR fluorescent probe **27** based on sulfur-substitution hemicyanine dye for H_2_S recognition was obtained by Su and colleagues. In contrast to traditional hemicyanine dyes, the oxygen in oxygen-substitution hemicyanine dyes was substituted by sulfur to become sulfur-substitution hemicyanine dyes. 2,4-dinitrophenyl served as the identifying site for H_2_S and the quenching group for probe fluorescence. As H_2_S concentration increases, the fluorescence signal around 787 nm was markedly increased (52-fold), red-shifted by 60 nm compared to oxygen-substituted hemicyanine dyes. As shown in Figure 9B, in the mouse model experiment of LPS-induced acute lung injury, the data showed a significant increase in H_2_S concentration.

### 3.6 Diabetic imaging

Diabetes, as a disease characterized by hyperglycemia, is related to diverse complications, including cardiovascular disease, stroke, kidney failure, neuropathy, retinopathy, and amputation (Al-Sofiani et al., 2019; Lau et al., 2019; Buades et al., 2021; Sempere-Bigorra et al., 2021; Milluzzo et al., 2021; O’neill et al., 2017). It is reported that diabetes can be divided into three types: Gestational diabetes, type 1 diabetes (T1D), and type 2 diabetes (T2D) (Xiang et al., 2018). H_2_S, as a promising candidate, helps to prevent and therapy of diabetes (Sun et al., 2021). Compared to lean participants, overweight and T2D patients had significantly lower blood levels of H_2_S (Whiteman et al., 2010). The protein expression and activity of CSE were significantly higher in peripheral blood mononuclear cells of normal humans than T1D patients (Manna et al., 2014). Therefore, studying the relationship between H_2_S and diabetes in-depth may be helpful to develop potential treatments for diabetes.

**FIGURE 10:**
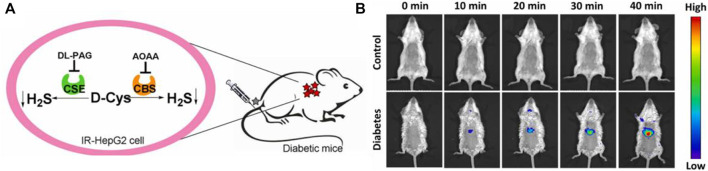
**(A)** Endogenous H_2_S biosynthesis in IR-HepG2 cells. **(B)** Fluorescence imaging of control (up) and diabetic (down) mice using probe 28 (reproduced from (Li Z et al., 2022) with permission from Elsevier (B. V).

In 2022, a “double-locked” fluorescent probe **28** with NIR emission for examining the H_2_S levels in organisms was obtained by Wei and colleagues. Probe **28** consisted of a fluorophore with NIR emission (rhodamine B), and re-active units of H_2_S (aromatic azide and NBD-piperazine). The fluorescence around 663 nm was locked and quenched through the intramolecular charge transfer (ICT) and photoinduced electron transfer (PET) processes. Probe **28** exhibited good selectivity and excellent sensitivity for imaging the behaviors of H_2_S. In addition, probe 28 was applied to image the levels of endogenous H_2_S in IR-Hepg2 cells and diabetic mice (Figure 10).

## 4 Summary and outlook

Fluorescence imaging may become a universally accepted diagnostic modality in the future due to its high efficiency and low cost. Accurate detection of H_2_S associated with pathophysiological processes and examining their behaviors are essential for understanding the diseases or pathophysiological processes involved, especially in the early stages. This paper reviews the bioimaging of H_2_S in pathophysiological processes (neurodegenerative diseases, inflammation, apoptosis, oxidative stress, organ injury, and diabetes) with fluorescent diagnostic probes. The design strategies, recognition mechanisms, optical properties, and applications of H_2_S fluorescent probes in bioimaging are further discussed. Up to now, remarkable progress has been achieved in exploring organic fluorescent probes for examining and studying H_2_S-associated pathophysiological processes in real-time.

Although delightful progress has been obtained, there are still some issues that need to be improved and solved: 1) Most fluorescent probes are inherently monochromatic, which can easily lead to false-positive signals in complex physiological settings, resulting in incorrect disease diagnosis; 2) Most H_2_S fluorescent probes reported to date have fluorescence emission wavelengths in the UV-visible region, which limits their application in studying diseases. There is still a large lack of H_2_S-based organic fluorescent probes that can be applied for routine diagnosis and monitoring of clinical diseases or pathophysiological processes. So it is crucial and urgent to construct novel fluorescent probes with fascinating advantages for imaging H_2_S associated with pathophysiological processes. To achieve this goal, we can start from the following aspects: 1) Designing fluorescent probes with excellent properties, including high quantum yields, large Stokes shifts, large photostability, and fast response; 2) Exploring the fluorescent probes of H_2_S with fine tissue penetration and high spatial resolution, which may have the greatest application due to the depth of biological tissues; 3) Developing organic fluorescent probes in the NIR-II region, which is expected to facilitate the development of systems suitable for monitoring deep organ-related diseases.

Overall, organic fluorescent probes with wonderful features might have the ability to image H_2_S associated with pathophysiological processes. It is believed that organic fluorescent probes for imaging H_2_S in pathophysiological processes will become increasingly vital testing tools in the future.
